# Sliding Mode Repetitive Control Based on the Unknown Dynamics Estimator of a Two-Stage Supply Pressure Hydraulic Hexapod Robot

**DOI:** 10.3390/biomimetics10070472

**Published:** 2025-07-18

**Authors:** Ziqi Liu, Bo Jin, Junkui Dong, Qingyun Yao, Yinglian Jin, Tao Liu, Binrui Wang

**Affiliations:** 1 College of Mechanical and Electrical Engineering, China Jiliang University, Hangzhou 310018, China; zqliu@cjlu.edu.cn; 2 State Key Laboratory of Fluid Power and Mechatronic Systems, Zhejiang University, Hangzhou 310027, China; bjin@zju.edu.cn (B.J.); liutao@zju.edu.cn (T.L.); 3 School of Mechanical Engineering, Zhejiang Sci-Tech University, Hangzhou 310018, China; dongjk@zstu.edu.cn; 4 Liangxin College, China Jiliang University, Hangzhou 310018, China; 2300102422@cjlu.edu.cn; 5 College of Modern Science and Technology, China Jiliang University, Hangzhou 310018, China; jinyinglian@cjlu.edu.cn

**Keywords:** hydraulic hexapod robot, sliding mode repetitive control, unknown dynamics estimator, adaptive robust sliding mode control

## Abstract

Hydraulic actuated legged robots display bright prospects and significant research value in areas such as unmanned area surveying, disaster rescue, military fields, and other scenarios owing to their excellent bionic characteristics, particularly their heavy payload capabilities and high power density. To realize the all-terrain adaptation locomotion of the hydraulic hexapod robot (HHR) with a heavy payload, one alternative control framework is position–posture control based on joint position control. As the foundation for the steady locomotion of HHRs, it is imperative to realize high-precision joint position control to improve the robustness under external disturbances during the walking process and to complete the attitude control task. To address the above issues, this paper proposes a sliding mode repetitive control based on the unknown dynamics estimator (SMRC + UDE) for the knee and hip joints of the HHR with a two-stage supply pressure hydraulic system (TSS). The effectiveness of the SMRC + UDE method is verified using a simulation environment and the ZJUHEX01 prototype experimental platform, and it is compared with the results for PID and adaptive robust sliding mode control (ARSMC). The results show that SMRC + UDE may be more suitable for our HHR, considering both the control performance and efficiency factors.

## 1. Introduction

Currently, bionic legged robots are a hot topic for researchers in carrying out scientific research work, including for use in imitating the galloping cheetahs to obtain high-speed locomotion [1], in challenging planetary analog environment exploration [2], for climbing on ferromagnetic surfaces [3], and for completing hydraulic legged mobile manipulators tasks [4]. Compared to wheeled or tracked robots, which rely on continuous contact with the ground, legged robots, especially quadruped and hexapod robots, possess superior mobility in challenging environments and uneven terrains via dynamically adjusting their leg movements and gaits. Owing to the urgent demand for both heavy payload and high-speed locomotion, many hydraulic quadruped robots and hydraulic hexapod robots (HHRs) have been developed and applied in payload transportation and unmanned surveying, including BigDog [5] from Boston Dynamics, the HyQ [6] series from the Italian Institutes of Technology (IIT), the SCalf [4] series from Shandong University, the hydraulic quadruped robot [7] from Yanshan University, COMET-IV [8] from Chiba University, HexaTerra [9] from the National Technical University of Athens, etc.

Efficient and steady locomotion control is the key for robots to realize various functions and complete operational tasks. A bionic hierarchical control architecture for legged robots was first proposed to travel in multiple terrains by Boston Dynamics in BigDog [5], in which it was applied and further developed. The hierarchical control architecture includes top-level, middle-level, and bottom-level control, analogous to the higher, lower, and motor nerves of legged animals in nature [10]. Based on the above idea of hierarchical control, HHRs typically utilize the following two motion control frameworks to realize all-terrain adaptation locomotion. One is similar to the dynamic model-based control in quadruped robots, including virtual model control (VMC) [11], model predictive control (MPC) [12], and whole-body control (WBC) [13], which is based on active compliance control. The other is position–posture control [14], which is based on joint position control. Since HHRs usually assume static gaits, such as a tripod or quadrangular gait, position–posture control adapted to complex terrains may be a suitable alternative; thus, high-precision inner-loop joint position control is necessary.

The joint position control of hydraulic legged robots is essentially the position closed-loop control of hydraulic actuators, mainly including proportional–integral–derivative (PID) control [15], active disturbance rejection control (ADRC) [16], feedback linearization (FL) control [17], robust and sliding mode control (SMC) [18], backstepping control [19], fuzzy control [20], adaptive control [21], neural network-based control [22], $H_{\infty }$ robust control [21], adaptive robust control (ARC) [23], and other control strategies. Cunha et al. [15] combined the gain algorithm with PID, and Focchi et al. [17] compared the performance of the linear quadratic regulator (LQR) controller, the feedback linearization (FL) controller, and PID in the HyQ leg prototype. Fan et al. [24] combined ADRC with the Lévy flight beetle antennae search algorithm (LBAS) to increase the robustness and control precision of a hydraulic quadruped robot. Shao et al. [25] used the self-growing Lévy flight salp swarm algorithm to find appropriate ADRC parameters and applied it in the joint system of a hydraulic quadruped robot. Zong et al. [26] proposed an ADRC strategy using double vane hydraulic rotary actuators for the hip joints of the quadruped robots to present better impact resistance ability. Zhu et al. [27] combined integral sliding mode control (ISMC) with state feedback $H_{\infty }$ control in the quadruped robot hydraulic drive unit (HDU). Gao et al. [22] designed a neural network (NN) model reference decoupling controller to reduce the influence of the coupling of the hydraulically quadruped robot. Zhou et al. [28] combined ARC with a constrained trajectory planner to realize the constrained motion control of an independent metering system.

In this paper, a sliding mode repetitive control based on the unknown dynamics estimator (SMRC + UDE) is designed for the joint position control of the independent metering hydraulic driving unit (IMHDU), which is used to drive the knee and hip joints of the hydraulic hexapod robot. The periodic joint trajectory is tracked via a sliding mode repetitive controller, while the lumped uncertain dynamic characteristics can be estimated via an unknown dynamics estimator. Simulations are carried out to prove that the SMRC + UDE strategy is stable and correct. PID, ARSMC, and SMRC + UDE are compared in the prototype experiments in terms of control performance and calculation efficiency. The results indicate that the SMRC + UDE controller exhibits superior joint control performance in the HHR among the three controllers, fulfilling the dual requirement of efficiency and accuracy.

The main contribution of this paper is to develop a sliding mode repetitive control strategy based on the unknown dynamics estimator (SMRC + UDE) for the IMHDU and apply it to the knee and hip joints of a hydraulic hexapod robot. This paper is constructed as follows. Section 2 provides an overview of the HHR, including its mechanical structure, control system architecture, and onboard hydraulic system. Section 3 presents the modeling process of the HHR, including the kinematics model, IMHDU model, straight gait planning, and foot trajectory planning. Section 4 describes the design implementations of SMRC + UDE, PID, and ARSMC for comparisons. The simulations and experiments are expressed and compared in Section 5. Finally, the conclusion is presented in Section 6.

## 2. Overview of Hydraulic Hexapod Robot

### 2.1. Mechanical Structure

Bio-inspired hydraulic multi-legged robots always imitate mammals or insects in nature. Since it is a hydraulic hexapod robot (HHR), there are two ways to configure its legs. Insect-inspired legged robots usually travel using a tripod gait or other static gaits, creating greater stability margins and load capacity, owing to their low center of gravity. However, they can only gain a slow speed of movement. Other mammal-inspired legged robots can achieve high dynamic motion with high agility, with this type mainly represented by quadruped robots.

**Figure 1:**
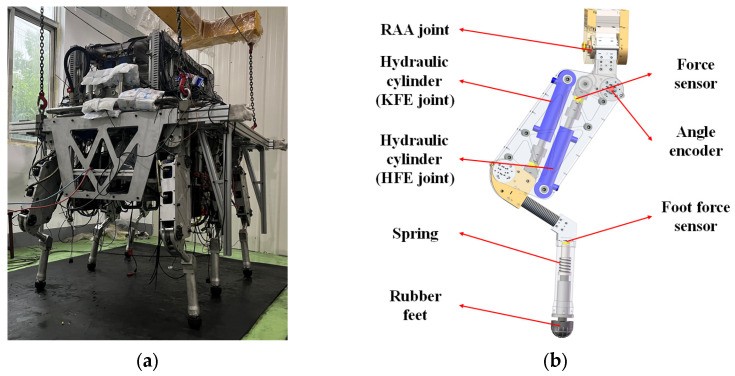
The hydraulic hexapod robot prototype ZJUHEX01 and its single-leg structure: (**a**) hydraulic hexapod robot prototype; (**b**) schematic diagram of single-leg structure.

Our proposed HHR ZJUHEX01 combines the consideration of movement capability, stability, and load capacity, which takes on a mammal-inspired configuration form, as shown in Figure 1. The HHR has a size of 1.65 m (length), 1.1 m (width), and 1.5 m (height), along with a body weight around of 800 kg and more than 300 kg of extra load capacity.

The proposed HHR adopts a bionic structure design comprising six mechanical legs and a trunk. Each mechanical leg imitates a mammal leg with three degrees of freedom (DOF), including root abduction/adduction (RAA), hip flexion/extension (HFE), and knee flexion/extension (KFE). Each DOF is actuated by a hydraulic cylinder, and the two hydraulic cylinders in the vertical plane are arranged in a cross pattern. This design draws on the biomechanical principle of “proximal mass concentration” to significantly increase the angular acceleration of the swing leg by reducing the shank inertia [29]. Meanwhile, the cross pattern reduces the joint angle range compared to that of the angular hydraulic cylinder arrangement of most hydraulic quadruped robots. Instead, it increases the hydraulic cylinder’s force arm, making it more suitable for large-load task operation scenarios. Furthermore, all six mechanical legs are configured in the form of the front knee, middle, and back elbow, enabling the HHR to simultaneously gain strong motion stability and large load capacity.

### 2.2. Control System Architecture

**Figure 2:**
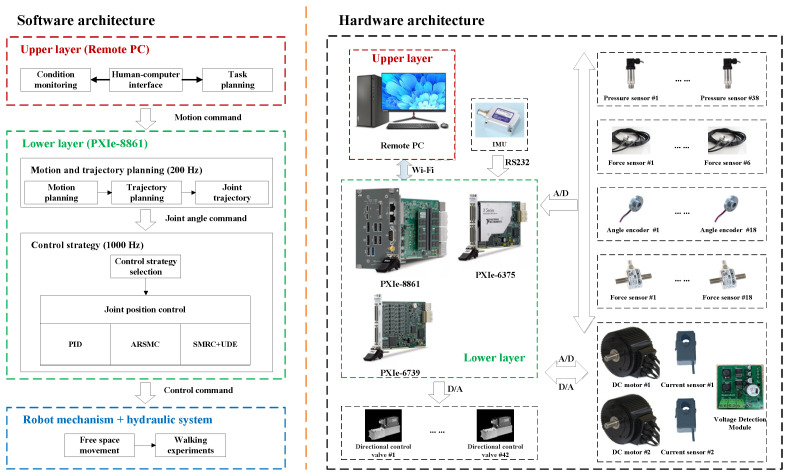
The hierarchical control system of the HHR.

As the mechanical structure of the legged robot is inspired by an animal, the control system architecture can also equally take a bionic design. To realize the HHR’s locomotion, a hierarchical control system inspired by higher animal motion control systems is established, as shown in Figure 2. The control system contains an upper-layer remote PC and a lower-layer industrial computer PXIe-8861 from National Instruments (NI). The upper layer remote PC is mainly responsible for condition monitoring, human–computer interface (HMI) interaction, and task planning. In contrast, the lower layer primarily comprises the PXIe-8861 controller, which contains a PXIe-6375 data acquisition card and a PXIe-6739 data output card. These two modules, respectively, handle multi-sensor signal acquisition and control signal output. Motion and trajectory planning of the HHR can be achieved at a 200 Hz frequency, while different control strategies can be computed at a 1000 Hz frequency in the lower layer. Owing to the coordination of the upper and lower layers, diverse motion and task functions of the HHR can be realized. More details about the control system’s operation principle and sensor specification have been illustrated in detail in the Refs. [30,31].

### 2.3. Onboard Hydraulic System

**Figure 3:**
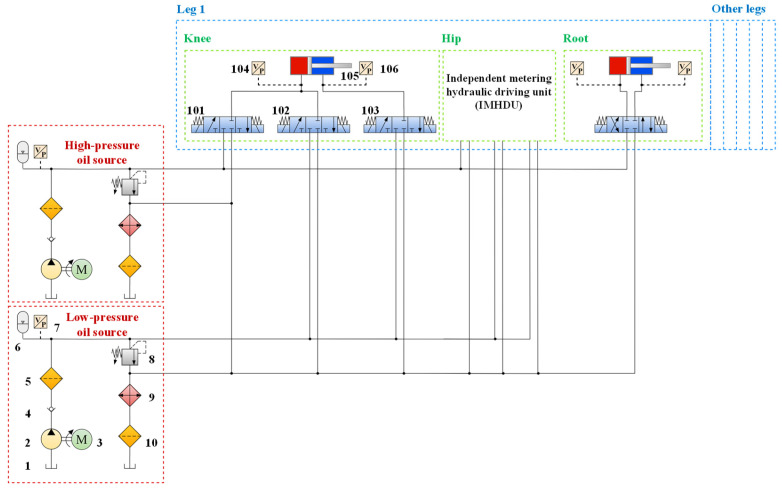
Schematic of the onboard two-stage supply pressure hydraulic system of the HHR: 1. oil tank; 2. low-pressure pump; 3. low-pressure motor; 4. check valve; 5. filter; 6. low-pressure accumulator; 7. pressure sensor; 8. relief valve; 9. oil cooler; 10. filter; 101. directional control valve $V_{v1}$; 102. directional control valve $V_{v2}$; 103. directional control valve $V_{v3}$; 104. pressure sensor; 105. hydraulic cylinder; 106. pressure sensor.

According to the principle of bionics, the energy supply system of a human or an animal includes an instantaneous energy system, a short-time energy system, and a long-time energy system, which generate the corresponding energy from anaerobic and aerobic metabolism, respectively. Drawing on this idea, an onboard bio-inspired two-stage supply pressure hydraulic system (TSS) is proposed in Figure 3. Specifically, a low-pressure oil source (18 MPa) with a high flow rate imitates the aerobic metabolism, which can maintain the low output power of the swing phase locomotion of the HHR. In comparison, a high-pressure oil source (6 MPa) with a low flow rate imitates anaerobic metabolism to supply the high output power of stance phase locomotion.

To meet the requirements of different pressure oil source switching, an independent metering hydraulic driving unit (IMHDU) is proposed, which is used in the knee joint and the hip joint. An independent metering system is also known as a separate meter-in and separate meter-out system (SMISMO), which eliminates the coupling relationship between the inlet and outlet ports of the two chambers of the hydraulic actuator and improves the degree of freedom of the system control compared with that of the traditional valve control system. In this way, hydraulic oil of different pressures can be fed into different chambers of each hydraulic cylinder to achieve energy-efficient locomotion. Specifications of the TSS schematic are illustrated in detail in Ref. [31].

## 3. HHR System Modeling

### 3.1. Kinematic Modeling

**Figure 4:**
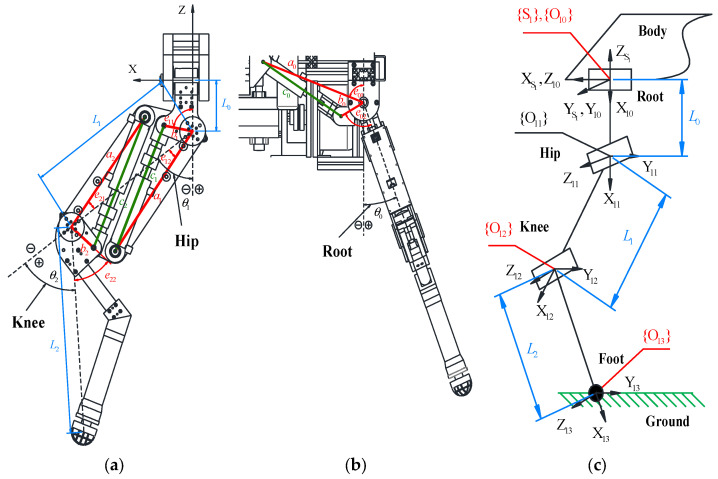
The structure and coordinate system of the single leg HHR: (**a**) the structure of the knee and hip joint; (**b**) the structure of the root joint; (**c**) the coordinate system of the leg.

Kinematic modeling is fundamental to motion control. For Leg 1 (left front leg), for instance, the geometric structure of the HHR single leg is shown in Figure 4. In the figure, $L_{0}$, $L_{1}$, and $L_{2}$ are the leg geometry parameters; $a_{0}$, $b_{0}$, $a_{1}$, $b_{1}$, $a_{2}$, $b_{2}$, $e_{11}$, $e_{12}$, $e_{21}$, and $e_{22}$ are the cylinder mounting position parameters; $\theta_{0}$, $\theta_{1}$, and $\theta_{2}$ are the joint angles of the root, hip, and knee, respectively.

According to the geometric relationship, the relationship between the length of the hydraulic cylinders and the joint angles can be shown as follows.

(1) $$\left\{\begin{array}{l} c_{0}=\sqrt{{a_{0}}^{2}+{b_{0}}^{2}+2a_{0}b_{0}\cos(\theta_{0}-e_{01}-e_{02})}=c_{00}+x_{pr}\\ c_{1}=\sqrt{{a_{1}}^{2}+{b_{1}}^{2}+2a_{1}b_{1}\cos(\theta_{1}-e_{11}+e_{12})}=c_{10}+x_{ph}\\ c_{2}=\sqrt{{a_{2}}^{2}+{b_{2}}^{2}+2a_{2}b_{2}\cos(\theta_{2}-e_{21}+e_{22})}=c_{20}+x_{pk} \end{array}\right.$$

where $c_{0}$, $c_{1}$, and $c_{2}$ represent the length of the hydraulic cylinders in the root, hip, and knee joint, respectively; $c_{00}$, $c_{10}$, and $c_{20}$ show the initial length of the hydraulic cylinders in the root, hip, and knee joint, respectively; $x_{pr}$, $x_{ph}$, and $x_{pk}$ represent the displacement of the hydraulic cylinder piston in the root, hip, knee joint, respectively.

The coordinate system of the single leg HHR is also shown in Figure 4c, in which $\left\{S_{1}\right\}$,$\left\{O_{10}\right\}$,$\left\{O_{11}\right\}$,$\left\{O_{12}\right\}$, and $\left\{O_{13}\right\}$ represent the leg base coordinate system, the root joint coordinate system, the hip joint coordinate system, the knee joint coordinate system, and the foot joint coordinate system, respectively. Using the Denavit–Hartenberg (D–H) method, the forward kinematics and inverse kinematics models are developed below:

(2) $$P(\theta )=\left[\begin{array}{c} -L_{1}\sin\theta_{1}-L_{2}\sin(\theta_{1}+\theta_{2})\\ (L_{0}+L_{1}\cos\theta_{1}+L_{2}\cos(\theta_{1}+\theta_{2}))\sin\theta_{0}\\ -(L_{0}+L_{1}\cos\theta_{1}+L_{2}\cos(\theta_{1}+\theta_{2}))\cos\theta_{0} \end{array}\right]=\left[\begin{array}{c} P_{x}\\ P_{y}\\ P_{z} \end{array}\right]$$

(3) $$\left\{\begin{array}{l} \theta_{0}=-\arctan\left(\frac{P_{y}}{P_{z}}\right)\\ \theta_{1}=-\arccos\left(\frac{{L_{1}}^{2}-{L_{2}}^{2}+L^{2}+P_{x}^{2}}{2L_{1}\sqrt{L^{2}+P_{x}^{2}}}\right)-\arctan\left(\frac{P_{x}}{L}\right)\\ \theta_{2}=\pi -\arccos\left(\frac{{L_{1}}^{2}+{L_{2}}^{2}-L^{2}-P_{x}^{2}}{2L_{1}L_{2}}\right) \end{array}\right.$$

where P(θ) is the foot position in the root joint coordinate system, and $L=\sqrt{P_{y}^{2}+P_{z}^{2}}-L_{0}$.

### 3.2. Independent Metering Hydraulic Driving Unit Modeling

**Figure 5:**
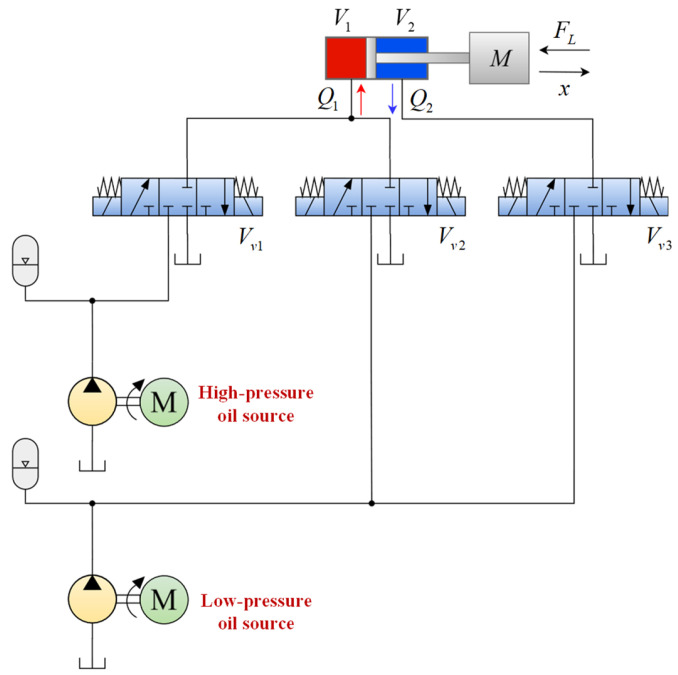
Schematic of the simplified knee joint IMHDU model.

Compared with the traditional valve-controller system, the IMHDU can separate the controls of the meter-in and meter-out orifices, which will increase the valve’s flexibility and the system’s energy efficiency. Considering the working conditions of different phases and leg configurations, both the hip and knee joints utilize IMHDU as the driving unit, while the root joint uses the traditional valve-controller system, as is shown in Figure 3. The simplified knee joint IMHDU model is established in Figure 5.

The pressure dynamics of the cylinder chambers, ignoring leakage, are derived by

(4) $$\frac{V_{1}}{\beta_{e}}{\dot{P}}_{1}=Q_{1}-A_{1}\dot{x}$$

(5) $$\frac{V_{2}}{\beta_{e}}{\dot{P}}_{2}=-Q_{2}+A_{2}\dot{x}$$

where $P_{1}$ and $P_{2}$ are the hydraulic cylinder pressure without the rod and with the rod, respectively; $V_{1}=V_{t1}+A_{1}x$, $V_{2}=V_{t2}-A_{2}x$, $V_{1}$, and $V_{2}$ are the hydraulic cylinder volume without the rod and with the rod, respectively; x represents the displacement of the hydraulic cylinder piston in the hip ($x_{ph}$) and knee joint ($x_{pk}$), respectively; $V_{t1}$ is the volume of the cavity between the valves and the hydraulic cylinders without the rod in the initial position; $V_{t2}$ is the volume of the cavity between the valves and the hydraulic cylinders with the rod in the initial position; $Q_{1}$ is the flow entering into the cylinder chamber without the rod; $Q_{2}$ is the flow leaving the cylinder chamber with the rod; $A_{1}$ and $A_{2}$ are the hydraulic cylinder areas without the rod and with the rod, respectively; $\beta_{e}$ is the bulk modulus of hydraulic oil.

Nonlinear functions are defined as

(6) $$g_{v1}(P_{1},sign(u_{i}))=\left\{\begin{array}{c} \sqrt{P_{s2}-P_{1}},u_{i}\geq 0\\ \sqrt{P_{1}-P_{r}},u_{i}<0 \end{array}\right.$$

(7) $$g_{v2}(P_{1},sign(u_{i}))=\left\{\begin{array}{c} \sqrt{P_{s1}-P_{1}},u_{i}\geq 0\\ \sqrt{P_{1}-P_{r}},u_{i}<0 \end{array}\right.$$

(8) $$g_{v3}(P_{2},sign(u_{i}))=\left\{\begin{array}{c} \sqrt{P_{s1}-P_{2}},u_{i}\geq 0\\ \sqrt{P_{2}-P_{r}},u_{i}<0 \end{array}\right.$$

where $P_{s1}$ and $P_{s2}$ are the low supply pressure and the high supply pressure; $P_{r}$ is the oil tank pressure; $u_{i}$ is the input voltage of the directional control valves.

A linear relationship between the input signal and the valve opening can be established. Thus, the flow functions can be written as

(9) $$Q_{v1}=k_{q}g_{v1}(P_{1},sign(u_{1}))u_{1}$$

(10) $$Q_{v2}=k_{q}g_{v2}(P_{1},sign(u_{2}))u_{2}$$

(11) $$Q_{v3}=-k_{q}g_{v3}(P_{2},sign(u_{3}))u_{3}$$

(12) $$\left\{\begin{array}{l} Q_{1}=Q_{v1}+Q_{v2}\\ Q_{2}=Q_{v3} \end{array}\right.$$

where $Q_{v1}$, $Q_{v2}$, and $Q_{v3}$ are the flow rate of three directional control valves $V_{v1}$, $V_{v2}$, and $V_{v3}$, respectively; $k_{q}$ is the valve’s flow gain coefficient.

The dynamics model of the hydraulic cylinder can be written as

(13) $$F_{cyl}=A_{1}P_{1}-A_{2}P_{2}$$

(14) $$F_{cyl}-F_{L}-F_{f}-B_{p}\cdot \dot{x}+\Delta =M\cdot \ddot{x}$$

where $F_{cyl}$ is the hydraulics cylinder output force; $F_{L}$ is the load force; $B_{p}$ is the viscous coefficient; M is the mass load; $F_{f}$ is the hydraulic cylinder friction force based on the Stribeck friction model, which can be estimated in the Ref. [32]; Δ is a concentrated uncertain nonlinearity of the system due to external disturbances, unmodeled friction, and difficult-to-model components.

As three valves are connected to a hydraulic cylinder to form an IMHDU, a proper valve configuration, shown in Table 1, is assumed to be given in case of conflicts. Owing to the foot force sensors installed on the robot’s feet, the ground reaction force (GRF) $G_{F}$ will be gained to easily distinguish whether the leg is in the stance phase or the swing phase. In the conventional one-stage supply pressure hydraulic system (OSS), only high pressure can be supplied, whether the leg is in the stance phase or the swing phase, which results in significant throttling loss in the low-load swing phase. On the contrary, in the TSS and IMHDU methods proposed, high-pressure and low-pressure can be switched appropriately according to the valve configuration; thus, the throttling loss will be significantly reduced, and energy will be saved.

### 3.3. Straight Gait Planning

Hexapod robots mimic the movement of multi-legged creatures, which can adopt multiple gaits, responding to different tasks and environments. The main periodic gaits of the hydraulic hexapod robot include the tripod, quadrangular, and pentagonal gaits. The most apparent difference between the three gaits is the number of stance legs and the duration of the stance legs. With the increase in the number of legs in the stance phase, the load capacity and the stability will also increase, while the locomotion speed will decrease. Thus, to balance the above three factors, the tripod and quadrangular gaits are mainly illustrated.

The duty factor (β) is the time fraction of a cycle when a particular leg is in the stance phase [33]. The duty factor can be written as

(15) $$\beta =\frac{t_{st}}{T_{cycle}}=\frac{t_{st}}{t_{st}+t_{sw}}$$

where $T_{cycle}$ is the duration of a gait cycle; $t_{st}$ and $t_{sw}$ are the duration when a particular leg is in the stance phase and the swing phase in a gait cycle.

**Figure 6:**
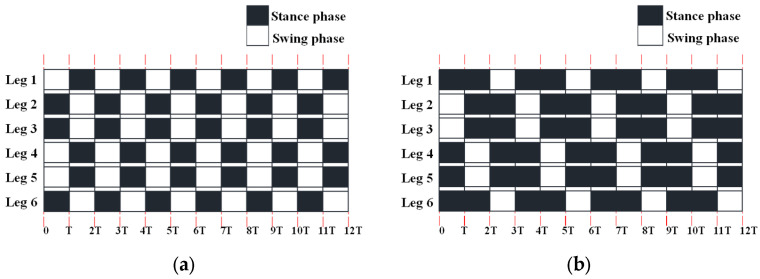
Time sequence diagram of the hydraulic hexapod robots walking forward in different gaits: (**a**) the tripod gait; (**b**) the quadrangular gait.

The duty factors of the tripod and quadrangular gaits are 1/2 and 2/3, respectively. The time sequence diagram of the hydraulic hexapod robot walking forward in the typical tripod and quadrangular gaits is shown in Figure 6. The shadowed area indicates that the particular leg is in the stance phase, while the white component represents the swing phase.

### 3.4. Foot Trajectory Planning

**Figure 7:**
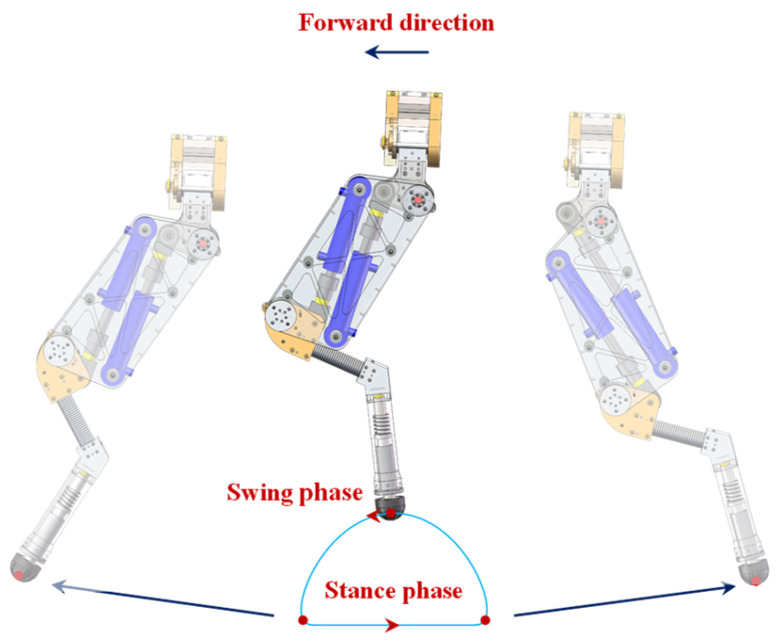
Foot trajectory in the XZ plane.

A suitable foot trajectory affects the speed and stability of robot locomotion. To realize the precise joint motion of the robot, it is necessary to fully consider the constraints of the robot’s feet to meet the requirements of trajectory smoothness and the continuity of velocity and acceleration. A sixth-order polynomial fitting curve is utilized to plan the foot trajectory, thereby reducing the impact of the foot when the phase is switching. The foot trajectory in the XZ plane is shown in Figure 7, where the HHR forward direction is from right to left. The parameters and the constraints of the foot trajectory can be seen in Ref. [30].

## 4. Highly Accurate Joint Position Controller Design

### 4.1. Problem Statement

A suitable locomotion control strategy is the key to smooth HHR motion. To realize the all-terrain adaptation locomotion of the HHR, one alternative control framework consists of position–posture control based on joint position control. Meanwhile, considering the characteristics of heavy load capacity (over 300 kg) when the HHR is walking on flat and structured ground, it is a natural idea to propose a highly accurate joint position controller with high stiffness to cope with external disturbances and shocks. It is a significant challenge for the controller design to deal with the time-variant joint load forces, nonlinearities in the hydraulic cylinders, uncertainties in the system model and parameters, shocks and vibrations from external disturbances, etc. Thus, in this section, three controllers are proposed and compared to realize highly accurate joint position control, including the proportional–integral–derivative (PID) controller, the adaptive robust sliding mode controller (ARSMC), and the sliding mode repetitive controller based on the unknown dynamic estimator (SMRC + UDE).

### 4.2. PID Controller

A PID controller can be described as

(16) $$u_{pid}=k_{p}e+k_{i}\int edt+k_{d}\frac{de}{dt}$$

(17) $$\left\{\begin{array}{l} u_{1}=u_{2}=u_{pid}\\ u_{3}=-u_{pid} \end{array}\right.$$

where *e* is the joint angle tracking error, $e=\theta_{d}-\theta$, $\theta_{d}$ is the reference angle, and θ is the actual angle; $k_{p}$, $k_{i}$, and $k_{d}$ are the proportional gain, integral gain, and derivative gain, respectively; $u_{1}$, $u_{2}$, and $u_{3}$ are the control voltage of valve $V_{v1}$, $V_{v2}$, and $V_{v3}$, respectively.

### 4.3. Adaptive Robust Sliding Mode Controller

**Figure 8:**
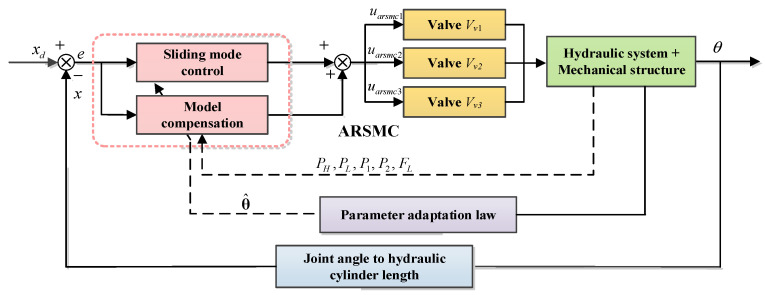
The schematic of ARSMC.

Adaptive robust control (ARC) is a control method that combines the advantages of adaptive control and robust control [34]. In the case of parameter uncertainty, the transient and steady-state errors can be controlled within a set of ranges through high-gain feedback. Combined with the sliding mode control (SMC) to form an adaptive robust sliding mode control (ARSMC), the original complex backstepping design process will be simplified to a feedback controller and sliding manifold design. The schematic of the ARSMC is illustrated in Figure 8.

According to Table 1, when the HHR is in the stance phase or swing phase, the corresponding valves will be configured. Assuming the leg is in the stance phase, only valve $V_{v1}$ and $V_{v3}$ are working; therefore, the control voltage $u_{1}$ and $u_{3}$ need to be designed. When the leg is in the swing phase, the control voltage $u_{2}$ and $u_{3}$ can be calculated similarly.

Based on Equations (4)–(14), the hydraulic cylinder dynamics can be written as

(18) $$\left\{\begin{array}{l} \frac{V_{1}}{\beta_{e}}{\dot{P}}_{1}=Q_{1}-A_{1}\dot{x}\\ \frac{V_{2}}{\beta_{e}}{\dot{P}}_{2}=-Q_{2}+A_{2}\dot{x}\\ F_{cyl}-F_{L}-F_{f}-B_{p}\cdot \dot{x}+\Delta =M\cdot \ddot{x}\\ F_{cyl}=A_{1}P_{1}-A_{2}P_{2} \end{array}\right.$$

Assuming that

(19) $$\left\{\begin{array}{l} Q_{1}={\tilde{Q}}_{1m}+Q_{1m}\\ Q_{2}={\tilde{Q}}_{2m}+Q_{2m} \end{array}\right.$$

where $Q_{1m}$ and $Q_{2m}$ are the nominal flow rate; ${\tilde{Q}}_{1m}$ and ${\tilde{Q}}_{2m}$ are the approximation error.

In general, the system is subjected to parametric uncertainties due to a lack of complete information. Thus, a set of parameters are defined as $\theta ={\left[\theta_{m},\theta_{d},\theta_{1m},\theta_{2m},\theta_{\beta_{e}},\theta_{B_{p}}\right]}^{T}$, $\theta_{m}=M$, $\theta_{d}=F_{L}+F_{f}$, $\theta_{1m}=\beta_{e}{\tilde{Q}}_{1m}$, $\theta_{2m}=\beta_{e}{\tilde{Q}}_{2m}$, and $\theta_{\beta_{e}}=\beta_{e}$, $\theta_{B_{p}}=B_{p}$. Let $\hat{\theta }$ denote the estimate of θ, and $\tilde{\theta }$ the estimation error (i.e., $\tilde{\theta }=\hat{\theta }-\theta$).

Equation (18) can be rewritten as

(20) $$\left\{\begin{array}{l} \ddot{x}=\frac{1}{\theta_{m}}(P_{1}A_{1}-P_{2}A_{2}-\theta_{B_{p}}\dot{x}-\theta_{d}+\Delta )\\ {\dot{P}}_{1}=\frac{\theta_{\beta_{e}}}{V_{1}}(Q_{1m}-A_{1}\dot{x}+\frac{\theta_{1m}}{\theta_{\beta_{e}}})\\ {\dot{P}}_{2}=\frac{\theta_{\beta_{e}}}{V_{2}}(A_{2}\dot{x}-Q_{2m}-\frac{\theta_{2m}}{\theta_{\beta_{e}}}) \end{array}\right.$$

**Assumption 1.**  *Parametric uncertainties and uncertain nonlinearities satisfy*

(21) $$\begin{array}{l} \theta \in \Omega_{\theta }≜\left\{\theta :\theta_{\min}<\theta <\theta_{\max}\right\}\\ \left|\Delta \right|\leq \delta_{d} \end{array}$$

*where* $\theta_{\min}$, $\theta_{\max}$, *and* $\delta_{d}$* are known.*

Firstly, the motion controller input $u_{1}$ can be designed as follows.

Define the tracking error as

(22) $$\left\{\begin{array}{l} e_{0}=\int \left(x-x_{d}\right)dt\\ e_{1}=x-x_{d}\\ e_{2}=\dot{x}-{\dot{x}}_{d}\\ e_{3}=\ddot{x}-{\ddot{x}}_{d} \end{array}\right.$$

An integral sliding manifold can be chosen as

(23) $$s=k_{0}e_{0}+k_{1}e_{1}+k_{2}e_{2}+e_{3}$$

where $k_{0}$, $k_{1}$, and $k_{2}$ can be chosen such that the polynomial $s^{3}+k_{2}s^{2}+k_{1}s+k_{0}$ is Hurwitz.

A positive semi-definite Lyapunov function can be chosen as

(24) $$V_{a1}=\frac{1}{2}\theta_{m}s^{2}$$

The derivative $V_{a1}$ can be expressed as

(25) $$\begin{array}{cc} {\dot{V}}_{a1}= & s[(\frac{A_{1}\theta_{\beta_{e}}}{V_{1}}Q_{1m}-\frac{{A_{1}}^{2}\theta_{\beta_{e}}}{V_{1}}\dot{x}-A_{2}{\dot{P}}_{2}+\frac{A_{1}\theta_{1m}}{V_{1}}-\theta_{B_{p}}\ddot{x}-{\dot{\theta }}_{d}+\Delta )\\ & -\theta_{m}{\dddot{x}}_{d}+\theta_{m}(k_{0}e_{1}+k_{1}e_{2}+k_{2}e_{3})] \end{array}$$

Now, design a virtual control law for $Q_{1m}$ as

(26) $$Q_{1m}=Q_{1ma}+Q_{1ms}=Q_{1ma}+Q_{1ms1}+Q_{1ms2}$$

(27) $$Q_{1ma}=\frac{V_{1}}{A_{1}}\frac{1}{{\hat{\theta }}_{\beta_{e}}}[\frac{{A_{1}}^{2}{\hat{\theta }}_{\beta_{e}}}{V_{1}}\dot{x}+A_{2}{\dot{P}}_{2}-\frac{A_{1}}{V_{1}}{\hat{\theta }}_{1m}+{\hat{\theta }}_{B_{p}}\ddot{x}+{\hat{\theta }}_{m}{\dddot{x}}_{d}-{\hat{\theta }}_{m}(k_{0}e_{1}+k_{1}e_{2}+k_{2}e_{3})]$$

(28) $$Q_{1ms1}=-\frac{V_{1}}{A_{1}}\frac{1}{\theta_{\beta_{e}\min}}s$$

(29) $$Q_{1ms2}=-\rho_{1}sign(s)$$

where $Q_{1ma}$ is the adaptive model compensation term; $Q_{1ms1}$ is the linear robust feedback term; $Q_{1ms2}$ is the nonlinear robust term, and $\rho_{1}\geq \rho_{01}=\frac{V_{1}}{A_{1}}\frac{1}{\theta_{\beta_{e}\min}}{\left|-{\dot{\theta }}_{d}+\Delta \right|}_{\max}$.

Thus, Equation (25) can be rewritten as

(30) $${\dot{V}}_{a1}=-\frac{\theta_{\beta_{e}}}{\theta_{\beta_{e}\min}}s^{2}-[\frac{A_{1}\theta_{\beta_{e}}}{V_{1}}\rho \left|s\right|-(-{\dot{\theta }}_{d}+\Delta )s]-\phi^{T}\tilde{\theta }s$$

where $\phi ={\left[-{\dddot{x}}_{d}+(k_{0}e_{1}+k_{1}e_{2}+k_{2}e_{3}),0,\frac{A_{1}}{V_{1}},0,-\frac{{A_{1}}^{2}}{V_{1}}\dot{x}+\frac{A_{1}}{V_{1}}Q_{1ma},-\ddot{x}\right]}^{T}$, τ=ϕs.

The adaptive law can be written as

(31) $$\dot{\hat{\theta }}={\mathrm{Proj}}_{\hat{\theta }}(\Gamma \tau )$$

(32) $${\mathrm{Proj}}_{{\hat{\theta }}_{i}}(\cdot i)=\left\{\begin{array}{l} 0,\;if{\hat{\theta }}_{i}=\theta_{i\max}\;and\cdot i>0\\ 0,\;if{\hat{\theta }}_{i}=\theta_{i\min}\;and\cdot i<0\\ \cdot i,\;otherwise \end{array}\right.$$

where Γ is a diagonal positive definite matrix; $\theta_{i\max}$ is the maximum value of the adaptive parameters; $\theta_{i\min}$ is the minimum value of the adaptive parameters.

Another positive semi-definite Lyapunov function combined with the adaptive law can be chosen as

(33) $$V_{A1}=V_{a1}+\frac{1}{2}{\tilde{\theta }}^{T}\Gamma^{-1}\tilde{\theta }=\frac{1}{2}\theta_{m}s^{2}+\frac{1}{2}{\tilde{\theta }}^{T}\Gamma^{-1}\tilde{\theta }$$

The derivative $V_{A1}$ can be expressed as

(34) $$\begin{array}{cc} {\dot{V}}_{A1} & ={\dot{V}}_{a1}+{\dot{\hat{\theta }}}^{T}\Gamma^{-1}\tilde{\theta }\\ & =-\frac{\theta_{\beta_{e}}}{\theta_{\beta_{e}\min}}s^{2}-[\frac{A_{1}\theta_{\beta_{e}}}{V_{1}}\rho \left|s\right|-(-{\dot{\theta }}_{d}+\Delta )s]+\tilde{\theta }({\dot{\hat{\theta }}}^{T}\Gamma^{-1}-\phi^{T}s)\\ & \leq 0 \end{array}$$

Thus, the designed controller is stable. Ignoring the dynamics of the valves, the control voltage $u_{1}$ can be calculated as follows:

(35) $$u_{1}=u_{arsmc1}=\frac{Q_{1m}}{k_{q}g_{v1}(P_{1},sign(u_{1}))}$$

Secondly, the control voltage $u_{3}$ can be designed similarly as follows:

(36) $$u_{3}=u_{arsmc3}=\frac{Q_{2m}}{k_{q}g_{v3}(P_{2},sign(u_{3}))}$$

where $Q_{2m}$ is a virtual control law analogous to $Q_{1m}$.

With the designed control voltage $u_{1}$ and $u_{3}$, the closed-loop system of IMHDU is stable, according to Lasalle’s invariant principle.

### 4.4. Sliding Mode Repetitive Controller Based on Unknown Dynamics Estimator

Although ARSMC has the advantages of higher accuracy and robustness, the shortcomings related to the huge amount of computation required are constantly revealed during the algorithm deployment in the experiments. Based on the TSS proposed in this paper, if the online calculation needs to be performed on six legs, with a total of 36 valves (except for the six root joint valves), the CPU will be occupied so heavily that it is difficult to run other algorithms subsequently.

As a result, a new idea was born. Why must we explicitly estimate all the unknown model parameters and uncertainties? The essence of the controller design is to improve control accuracy, not to calculate all unknown quantities. Suppose that the unknown model parameters and uncertainties are estimated as a whole and applied as an equivalent nonlinear compensation in the control voltage. Will the accuracy also be improved, to some extent?

**Figure 9:**
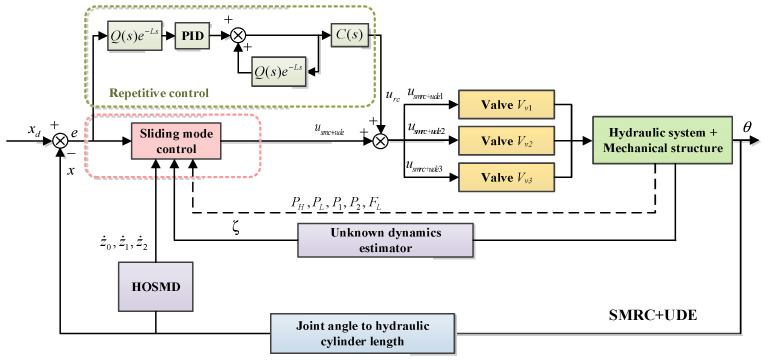
The schematic of SMRC + UDE.

For this reason, a sliding mode repetitive control based on the unknown dynamics estimator (SMRC + UDE) is proposed to track the periodic joint trajectory and improve the control accuracy. On the one hand, the unknown dynamics estimator (UDE) deals with the lumped uncertain dynamic characteristics, such as the nonlinear and unmeasurable dynamics that cannot be modeled. On the other hand, considering the regular gait’s periodicity and the joint trajectory’s repetitiveness, the advantages of the repetitive control (RC) are combined with those of the proposed controller to form a sliding mode repetitive controller (SMRC). The schematic of the SMRC + UDE is illustrated in Figure 9.

Firstly, a sliding mode control based on the unknown dynamics estimator (SMC + UDE) is proposed to realize accurate joint trajectory tracking. Similarly, the case of the stance phase is considered. The control voltage $u_{1}$ and $u_{3}$ are designed as follows.

Firstly, the motion controller input $u_{1}$ can be designed as follows:

Define the state variables $x={\left[x_{1},x_{2},x_{3},x_{4}\right]}^{T}={\left[x,\dot{x},\ddot{x},F_{cyl}\right]}^{T}$, and let $d=F_{L}+F_{f}-\Delta$; the entire system can be expressed in a state-space form as

(37) $$\left\{\begin{array}{l} {\dot{x}}_{1}=x_{2}\\ {\dot{x}}_{2}=x_{3}=g_{1}x_{4}+f_{1}-g_{1}d\\ {\dot{x}}_{3}=g_{1}(g_{21}u_{1}+f_{21}-A_{2}{\dot{P}}_{2})+{\dot{f}}_{1}-g_{1}\dot{d} \end{array}\right.$$

where a set of new parameters can be defined as

(38) $$\left\{\begin{array}{l} f_{1}=-\frac{B_{p}}{M}\dot{x}\\ g_{1}=\frac{1}{M}\\ f_{2}=-\left(\frac{{A_{1}}^{2}}{V_{1}}+\frac{{A_{2}}^{2}}{V_{2}}\right)\beta_{e}\dot{x}\\ f_{21}=-\frac{{A_{1}}^{2}}{V_{1}}\beta_{e}\dot{x}\\ f_{22}=-\frac{{A_{2}}^{2}}{V_{2}}\beta_{e}\dot{x}\\ g_{2}=\left[\frac{A_{1}}{V_{1}}g_{v1}(P_{1},sign(u_{1}))+\frac{A_{2}}{V_{2}}g_{v3}(P_{2},sign(u_{3}))\right]\beta_{e}k_{q}\\ g_{21}=\frac{A_{1}}{V_{1}}g_{v1}(P_{1},sign(u_{1}))\beta_{e}k_{q}\\ g_{22}=\frac{A_{2}}{V_{2}}g_{v3}(P_{2},sign(u_{3}))\beta_{e}k_{q} \end{array}\right.$$

In Ref. [30], a high-order sliding mode differentiator (HOSMD) is proposed to gain the joint angular velocity and acceleration. Thus, the HOSMD can also be utilized to observe the state variables $x_{2}$ and $x_{3}$. Define the new state variables as $z_{0}=x_{1}$, $z_{i}={\dot{z}}_{i-1}$, i=1,2. The system state equations can be written in a Brunovsky canonical form.

(39) $$\left\{\begin{array}{l} {\dot{z}}_{0}=z_{1}\\ {\dot{z}}_{1}=z_{2}\\ {\dot{z}}_{2}=g_{1}(g_{21}u_{1}+f_{21}-A_{2}{\dot{P}}_{2})+{\dot{f}}_{1}-g_{1}\dot{d}=\zeta_{1}(x_{2},x_{3})+p_{1}u_{1} \end{array}\right.$$

where $\zeta_{1}(x_{2},x_{3})$ is the unknown lumped dynamics, $\zeta_{1}(x_{2},x_{3})=g_{1}f_{21}-g_{1}A_{2}{\dot{P}}_{2}+{\dot{f}}_{1}-g_{1}\dot{d}$;$p_{1}=g_{1}g_{21}$.

**Assumption 2.**  *The derivative of the system unknown lumped dynamics is bounded.*

(40) $$\sup_{t\geq 0}\left|{\dot{\zeta }}_{1}\right|\leq ƛ,\exists ƛ>0$$

This assumption is the same as that required in the designs of ESO in ADRC [16], which can be fulfilled in practice.

Considering Equation (39), a first-order low-pass filter can be used to smooth the variables $z_{2}$ and $u_{1}$ as

(41) $$\left\{\begin{array}{l} T_{s}{\dot{z}}_{2f}+z_{2f}=z_{2},z_{2f}(0)=0\\ T_{s}{\dot{u}}_{1f}+u_{1f}=u_{1},u_{1f}(0)=0 \end{array}\right.$$

where $T_{s}>0$ is the designed filter parameter.

**Lemma 1.**  *Considering the system Equation (39) and the filter Equation (41), let an auxiliary variable* $\beta =\frac{\left(z_{2}-z_{2f}\right)}{T_{s}}-p_{1}u_{1f}-\zeta_{1}$ *be bounded and decrease to a small residual set exponentially for* $T_{s}>0$ *Hence, the manifold* β=0 *is an invariant manifold for* $T_{s}\to 0$ *.*

(42) $$\beta =\frac{\left(z_{2}-z_{2f}\right)}{T_{s}}-p_{1}u_{1f}-\zeta_{1}=0$$

The proof of Lemma 1 is given in Appendix A. Hence, the unknown lumped dynamics $\zeta_{1}$ can be estimated as

(43) $$\zeta_{1}=\frac{\left(z_{2}-z_{2f}\right)}{T_{s}}-p_{1}u_{1f}$$

Similarly, the tracking error and the integral sliding manifold can be chosen as Equations (22) and (23).

Another positive semi-definite Lyapunov function can be chosen as

(44) $$V_{s1}=\frac{1}{2}s^{2}$$

The derivative $V_{s1}$ can be expressed as

(45) $${\dot{V}}_{s1}=s\dot{s}=s(k_{0}e_{1}+k_{1}e_{2}+k_{2}e_{3}+{\dot{e}}_{3})=s(k_{0}e_{1}+k_{1}e_{2}+k_{2}e_{3}+\zeta_{1}+p_{1}u_{1}-{\dddot{x}}_{d})$$

The control voltage $u_{1}$ of SMC + UDE can be designed as

(46) $$u_{1}=u_{smc+ude1}=-\frac{\left(k_{0}e_{1}+k_{1}e_{2}+k_{2}e_{3}+\zeta_{1}-{\dddot{x}}_{d}\right)}{p_{1}}-\rho sign(s)$$

where $\rho \geq \rho_{0}>0$.

Thus

(47) $${\dot{V}}_{s1}=-\rho \left|s\right|\leq 0$$

Similarly, the control voltage $u_{3}$ of SMC + UDE can be designed as

(48) $$u_{3}=u_{smc+ude3}=-\frac{\left(k_{0}e_{1}+k_{1}e_{2}+k_{2}e_{3}+\zeta_{3}-{\dddot{x}}_{d}\right)}{p_{3}}-\rho sign(s)$$

where $p_{3}=g_{1}g_{22}$.

With the designed control voltage $u_{1}$ and $u_{3}$, the closed-loop system of IMHDU is stable according to Lasalle’s invariant principle. As the changes in the model parameters are considered as the unknown lumped dynamics, when it comes to the experiments, we only need to tune the controller parameters $k_{0}$, $k_{1}$, $k_{2}$, β, and the estimator parameter $T_{s}$ to proper values to maintain the robustness.

Secondly, considering the repetitive and periodic characteristics of the joint trajectories, combining the advantages of repetitive control (RC) can improve its control performance. The schematic RC is illustrated as a part of SRMC + UDE in Figure 9.

The transfer function from $u_{rc}$ to the error e is shown as follows:

(49) $$G_{rc}(s)=\frac{Q(s)e^{-Ls}G_{PID}(s)C(s)}{1-Q(s)e^{-Ls}}$$

where $G_{rc}(s)$ is the transfer function from position error *e* to RC output $u_{rc}$; Q(s) is the compensation term to ensure system stability, which is always chosen as a constant of nearly 1 or a low-pass filter; $e^{-Ls}$ is the time delay element, *L* is the delay time, which can be chosen consistent with the control frequency; $G_{PID}(s)$ is the transfer function of the PID controller; C(s) is the stabilization compensation term for the amplitude and phase correction of the controller.

Overall, the final control voltage $u_{1}$ and $u_{3}$ of SMRC + UDE can be written as follows:

(50) $$u_{1}=u_{smrc+ude1}=u_{smc+ude1}+u_{rc}$$

(51) $$u_{3}=u_{smrc+ude3}=u_{smc+ude3}+u_{rc}$$

Also, when the leg is in the swing phase, the control voltage $u_{2}$ and $u_{3}$ can be calculated similarly.

## 5. Simulation and Experiment

Two error indices are used to evaluate the control responses of different controllers and analyze the control performance quantitatively.

1. The maximum value of the absolute error (MAXE).

(52) $$\mathrm{MAXE}=\max\left\{\left|\theta_{d}(i)-\theta (i)\right|\right\}$$

2. The root mean square error (RMSE).

(53) $$\mathrm{RMSE}=\sqrt{\frac{1}{N}\sum_{i=1}^{N}{\left(\theta_{d}(i)-\theta (i)\right)}^{2}}$$

where $\theta_{d}$ is the reference angle; θ is the actual angle; *i* is the *i*th data of the angles.

### 5.1. MATLAB Simulink and ADAMS Co-Simulation

**Figure 10:**
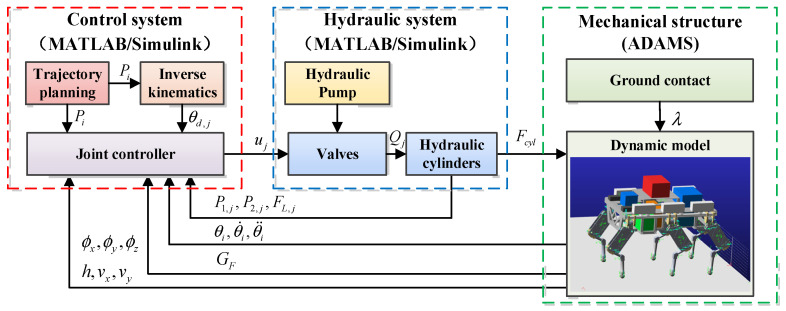
The schematic of the co-simulation model of the HHR.

A co-simulation model based on MATLAB Simulink (R2017a) and ADAMS 2015 is established to verify the validity of the proposed controllers. As is shown in Figure 10, the control system and the hydraulic system are established in MATLAB/Simulink, while the mechanical structure is built in ADAMS. This co-simulation model integrates the advantages of numerical simulation and the characteristics of the HHR dynamics model, which will provide a realistic simulation of the hydraulic dynamics and control performance. The details of different components in the co-simulation model are shown in Ref. [30].

**Figure 11:**
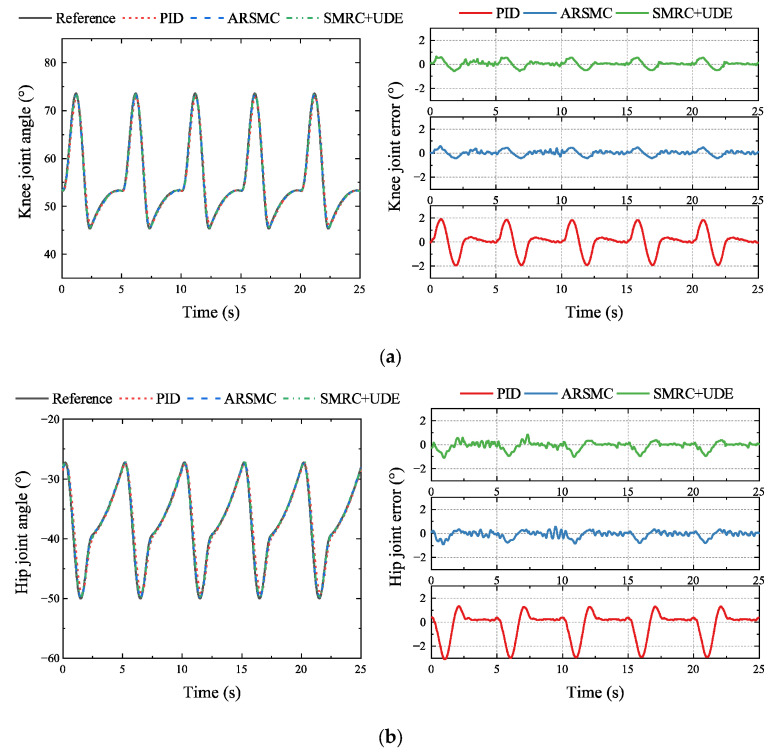
Comparison of PID, ARSMC, and SMRC + UDE regarding joint trajectory tracking and errors in the simulation: (**a**) knee trajectory and tracking error; (**b**) hip trajectory and tracking error.

In the simulation, the locomotion performance of the HHR walking in a tripod gait was tested. Three controllers, including PID, ARSMC, and SMRC + UDE, were implemented in the knee and hip joint, and the angle trajectory and tracking errors are shown in Figure 11.

It was obvious that ARSMC and SMRC + UDE could significantly improve the control accuracy. Compared with the PID controller, the MAXE of the knee and hip joints using ARSMC could reach 0.58° and 0.92°, which could be reduced by 70.44% and 70.24%, respectively. Compared with the PID controller, the MAXE of the knee and hip joints using SMRC + UDE could reach 0.64° and 1.10°, which could be reduced by 70.35% and 64.19%, respectively. The above results showed that the proposed control algorithms, ARSMC and SMRC + UDE, were initially effective.

### 5.2. Prototype Experiment

#### 5.2.1. Single-Leg Joint Control Experiment

Before the walking experiments, the single-leg joint control experiment needed to be carried out. As the root joint had a small range of motion, the PID controller was implemented. The knee joint and the hip joint mainly participated in the motion when the HHR was walking straight; thus, different control performances of PID, ARSMC, and SMRC + UDE were compared in these two joints. Two experiments were conducted in the single-leg joint control tests, including E1 and E2. In E1, the 1 Hz sinusoidal signal was implemented in two joints, while in E2, the 0.5 Hz joint trajectory generated by inverse kinematics was implemented. In experiments E1 and E2, HHR was placed on a support frame without contact with the ground, and only Leg 1 was controlled for the movement. Thus, a ground reaction force signal must be given to realize the valve switching of IMHDU.

1. Experiment E1

The knee trajectory to be tracked was $\theta_{dk}=(180/\pi )[1+0.2\sin(2\pi t)]$(°). The hip trajectory to be tracked was $\theta_{dh}=(180/\pi )[-0.5+0.2\sin(2\pi t)]$(°). The simulated ground reaction force signal was $G_{F}=[100\sin(2\pi t)+100]$(N). When $G_{F}\geq 100$, the stance phase was simulated with the valves $V_{v1}$ and $V_{v3}$ turned on, and when $G_{F}<100$, the swing phase was simulated with the valves $V_{v2}$ and $V_{v3}$ turned on, as shown in Table 1.

2. Experiment E2

As the joint trajectory obtained from the inverse kinematics solution of the foot trajectory in Figure 7 was not a simple sinusoidal signal, it was difficult to illustrate the superiority of the proposed controller using only the sinusoidal tracking. Thus, it was necessary to compare the three proposed controllers with the joint motion trajectory. In this experiment, a 0.5 Hz joint motion trajectory was tested, equivalent to the HHR moving at the maximum speed of 0.375 m/s, in order to test the locomotion ability of the hydraulic actuated joints. The simulated ground reaction force signal was $G_{F}=[100\sin(\pi t)+100]$ (N).

**Figure 12:**
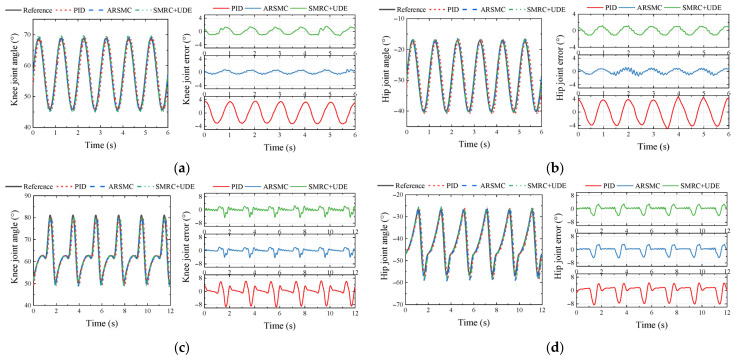
Comparison of PID, ARSMC, and SMRC + UDE in 1 Hz sinusoidal signal (E1) and 0.5 Hz joint motion trajectory (E2) tracking and errors: (**a**) knee trajectory in 1 Hz sinusoidal signal; (**b**) hip trajectory in 1 Hz sinusoidal signal; (**c**) knee trajectory in 0.5 Hz joint motion trajectory signal; (**d**) hip trajectory in 0.5 Hz joint motion trajectory signal.

The main parameters of the controllers are illustrated in detail in Appendix B. The results of E1 and E2 are shown in Figure 12, which visually compares three control algorithms. Similar to the co-simulation results, it was evident that ARSMC and SMRC + UDE could significantly improve the control accuracy, while ARSMC exhibited the highest control accuracy among the three. Meanwhile, from Figure 12a,b, the MAXE of the knee and hip joints using SMRC + UDE could reach 1.55° and 1.21° in E1, while from Figure 12c,d, the MAXE results reached 4.78° and 5.83° in E2. It was found that the proposed controllers could achieve better tracking accuracy via a simple 1 Hz sinusoidal signal input (E1) than using the 0.5 Hz joint trajectory signal (E2) with the same control parameters. In order to determine the reason for the deterioration in control performance from E1 to E2, a fast Fourier transform (FFT) was conducted. The FFT results showed that the 0.5 Hz joint trajectory (E2) contained multiple high-frequency sinusoidal signals above 1 Hz superimposed on each other, thereby affecting the control performance. This phenomenon will subsequently guide the parameter tuning.

#### 5.2.2. Dilemma Between Control Performance and Efficiency

**Figure 13:**
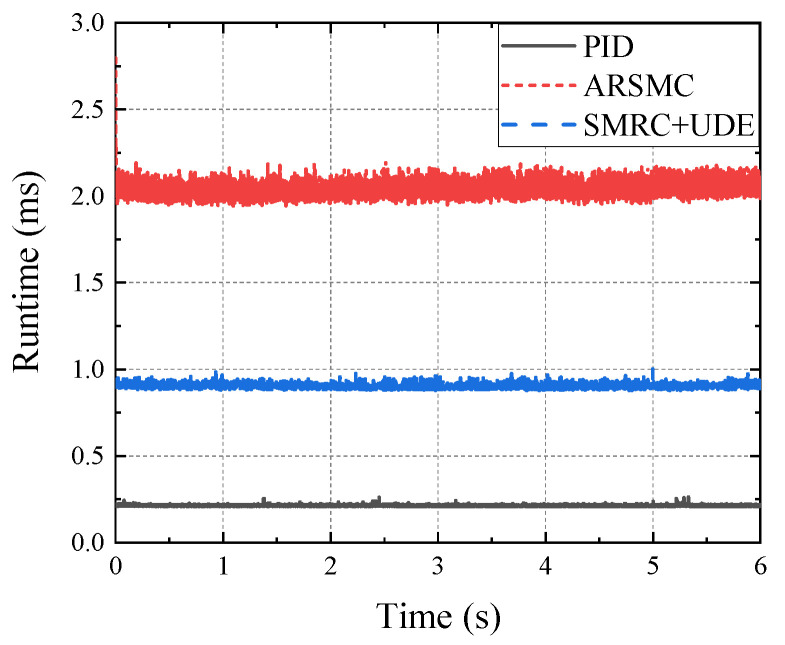
Comparison of CPU runtime occupied by PID, ARSMC, and SMRC + UDE in E2.

From Figure 12, ARSMC is observed to show the highest control accuracy among the three control algorithms in the single-leg joint control experiments (only six valves). However, considering the control system architecture, the control algorithms calculated in the lower layer will occupy less than 1 ms of runtime, as the control frequency is set to 1000 Hz for the position and force closed-loop control. For this reason, we tested the runtimes of three control algorithms when they were deployed to 36 valves on all six legs, as shown in Figure 13. Furthermore, we compared PID, ARSMC, and SMRC + UDE quantitatively in E2, along with control performance and algorithm efficiency, as shown in Table 2.

Consistent with the theoretical analysis, we are caught in a dilemma between control performance and efficiency. On the one hand, ARSMC displays higher control accuracy in $\theta_{1}$ and $\theta_{2}$ but requires a 2.04 ms runtime, far beyond the control cycle (1 ms). As ARSMC requires extensive online parameter estimation, it improves the control accuracy at the expense of efficiency. On the other hand, SMRC + UDE is slightly inferior in regards to accuracy when compared to that of ARSMC but requires a 0.90 ms runtime, with higher efficiency. In such a dilemma, to subsequently add more complex algorithms in IMHDU and to avoid the program runaway, SMRC + UDE may be more suitable for meeting the requirements for achieving efficient and accurate joint control of the HHR system in walking experiments E3 and E4.

#### 5.2.3. Walking Experiment

In the walking experiments, E3 quadrangular gait and E4 tripod gait were assessed to validate the effectiveness of the proposed SMRC + UDE.

1. Experiment E3

**Figure 14:**
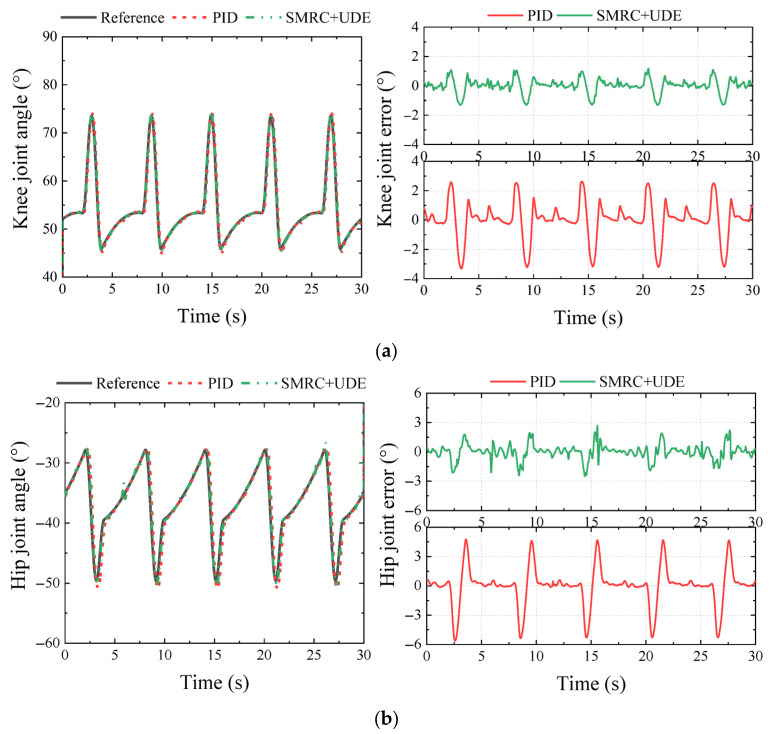
Comparison of PID and SMRC + UDE in 0.17 Hz quadrangular gait walking experiment (E3): (**a**) knee trajectory and tracking error; (**b**) hip trajectory and tracking error.

E3 evaluated a 0.17 Hz quadrangular gait of the HHR. Compared to the tripod gait, the speed of the quadrangular gait was relatively slower, but it offered a greater margin of stability, better stabilization, and heavier load capacity. The angle trajectory and tracking errors are shown in Figure 14.

Figure 14 shows that SMRC + UDE displayed smaller tracking errors in the knee and hip joints. In the knee joint, the MAXE under SMRC + UDE and PID were 1.31° and 3.31°, respectively, representing an error reduction of 60.6%. In the hip joint, the MAXE under SMRC + UDE and PID were 2.68° and 5.62°, respectively, representing an error reduction of 52.3%.

2. Experiment E4

**Figure 15:**
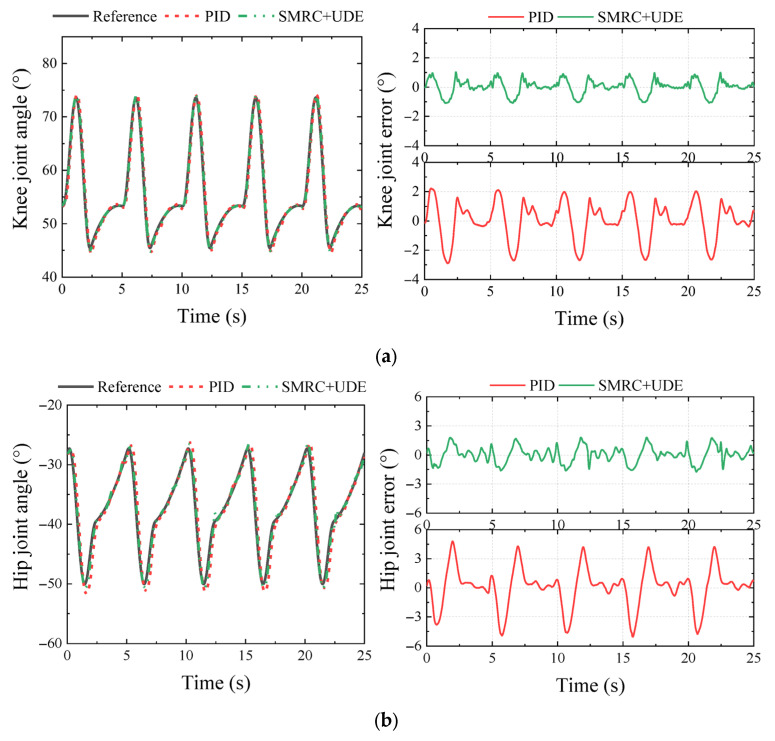
Comparison of PID and SMRC + UDE in 0.2 Hz tripod gait walking experiment (E4): (**a**) knee trajectory and tracking error; (**b**) hip trajectory and tracking error.

**Figure 16:**
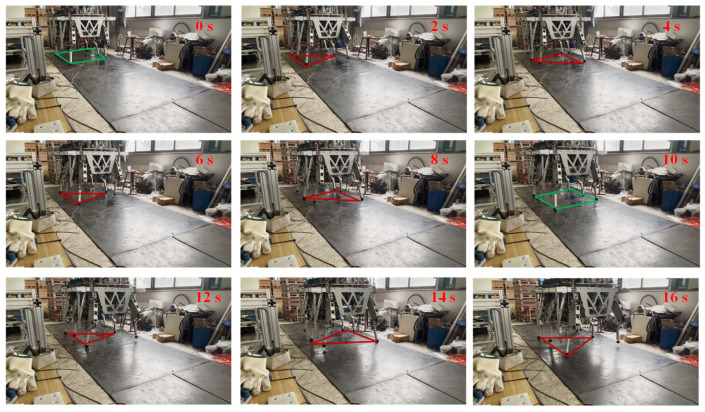
Snapshot of the HHR walking in a tripod gait in 0–16 s.

E4 evaluated a 0.2 Hz tripod gait of the HHR. The angle trajectory and tracking errors are shown in Figure 15. The snapshot of the E4 in 0–16 s is shown in Figure 16, where the red triangles in the figure represent the support triangle of the stance legs in the tripod gait, and the green quadrangles indicate the support polygon of all six stance legs.

From Figure 15, the effectiveness of SMRC + UDE can also be verified. In the knee joint, the MAXE under SMRC + UDE and PID were 1.10° and 2.90°, respectively, representing an error reduction of 62.1%. In the hip joint, the MAXE under SMRC + UDE and PID were 1.79° and 5.04°, respectively, representing an error reduction of 64.2%.

#### 5.2.4. Data Analysis

**Figure 17:**
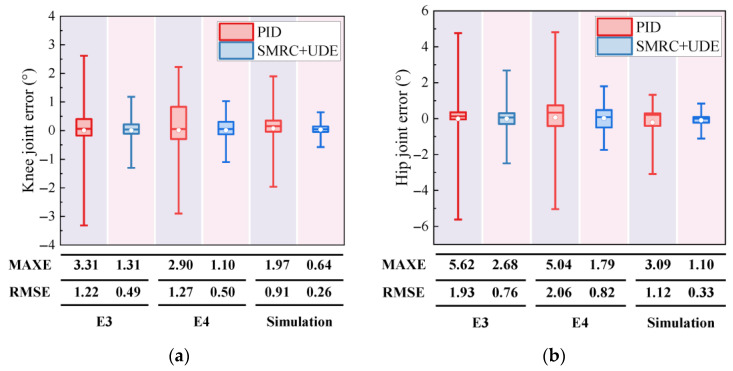
Joint errors of PID and SMRC + UDE in E3, E4, and the simulation: (**a**) knee joint error; (**b**) hip joint error.

To further show the effectiveness of SMRC + UDE, two error indices, MAXE and RMSE, are illustrated in Figure 17. The results of E3, E4, and the simulation provide a quantitative comparison of PID and SMRC + UDE.

For example, the knee joint error of PID showed a MAXE of 3.31° in E3, 2.90° in E4, and 1.97° in the simulation. For the hip joint, the MAXE of PID was 5.62° in E3, 5.04° in E4, and 3.09° in the simulation. Concerning the proposed SMRC + UDE, the knee joint error showed a MAXE of 1.31° in E3, 1.10° in E4, and 0.64° in the simulation. For the hip joint, the MAXE was 2.68° in E3, 1.79° in E4, and 1.10° in the simulation. Compared with PID, the knee joint error could be reduced by 60.6% in E3, 62.1% in E4, and 70.35% in the simulation, while the hip joint error could be reduced by 52.3% in E3, 64.2% in E4, and 64.19% in the simulation, respectively. Furthermore, the RMSE of the knee joint could reach 0.49° in E3, 0.50° in E4, and 0.26° in the simulation, while the RMSE of the hip joint could reach 0.76° in E3, 0.82° in E4, and 0.33° in the simulation.

In summary, whatever state the joint IMHDU actuator system was in, the proposed SMRC + UDE controller showed significant improvements in control performance compared with the results for PID. Currently, most hydraulic driving units (HDUs) for hydraulic quadruped robots or hexapod robots utilize expensive servo valves. Our proposed SMRC + UDE can also achieve a similar control performance, using only the industrial directional control valves, which can significantly reduce the manufacturing costs and increase the possibility of its application in hydraulic legged robots.

**Figure 18:**
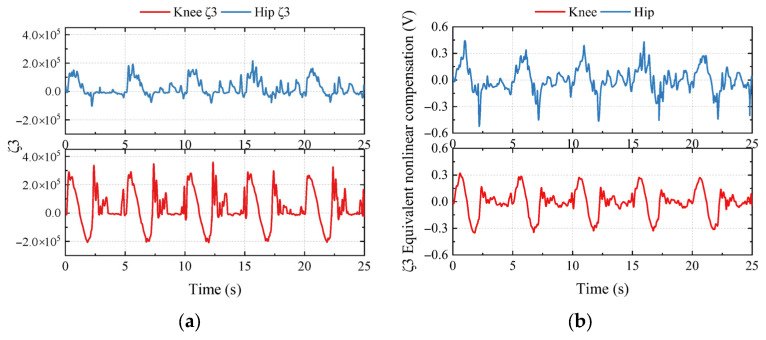
The estimator results of $\zeta_{3}$ and its equivalent nonlinear compensation term: (**a**) the estimator result of $\zeta_{3}$; (**b**) the equivalent nonlinear compensation term of $\zeta_{3}$.

To determine how the UDE works, the unknown lumped dynamics ζ was recorded. According to Table 1, the valve $V_{v1}$ and $V_{v2}$ in IMHDU switched frequently to supply different stage pressures, while the valve $V_{v3}$ was working constantly, whether in the swing phase or stance phase. Thus, the unknown lumped dynamics $\zeta_{3}$ could be estimated and transferred to the nonlinear compensation term in the control voltage, according to Equation (A1). Figure 18 shows the estimator results of $\zeta_{3}$ and its equivalent nonlinear compensation term.

**Figure 19:**
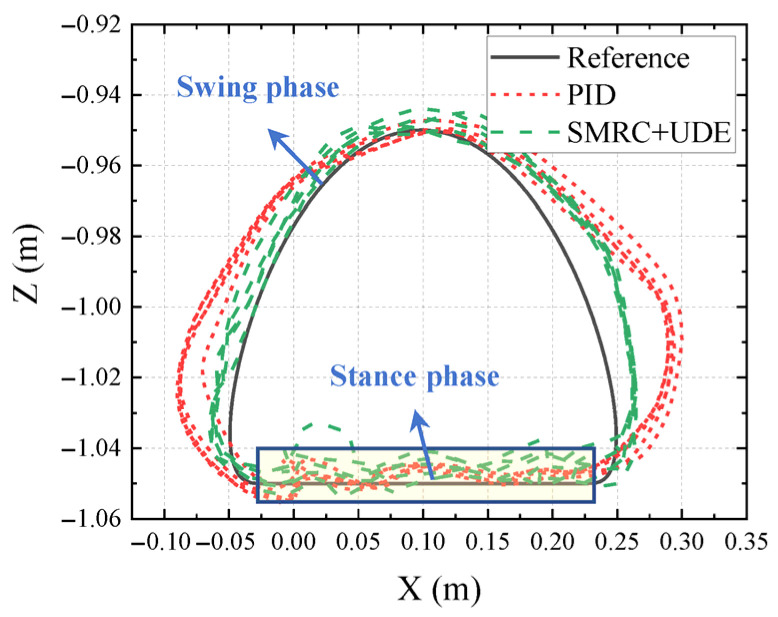
Foot trajectory of Leg 1 in the XZ plane.

In addition to the joint tracking, the foot trajectory of Leg 1 in the XZ plane could be calculated with forward kinematics, as shown in Figure 19. The improvement in the joint trajectory tracking in the SMRC + UDE could be displayed by the tracking errors in the X and Y directions when the leg was in the swing phase, which showed smaller deviations from the reference. However, when the leg was in the stance phase, the foot trajectory of both PID and SMRC + UDE showed a certain degree of shock and oscillation. Meanwhile, it was determined that, although E4 gained better tracking performance than did E3, the snapshot of E3 demonstrated better motion stability and less shaking of the HHR than did E4. These two phenomena may be related to the interaction between the robot’s feet and the outside environment. The question of how to balance the relationship between robot joint control and body motion urgently needs to be followed up in subsequent research.

## 6. Conclusions

This paper focused on the control of the IMHDU in a two-stage supply pressure hydraulic hexapod robot, and an accurate and efficient sliding mode repetitive control based on the unknown dynamics estimator (SMRC + UDE) was proposed. The proposed method utilized SMRC to track periodic joint trajectory, while using UDE to deal with the lumped uncertain dynamic characteristics, achieving a highly accurate joint position control. Both a simulation and experiments were carried out to prove the effectiveness of this proposed method. The main conclusions are summarized as follows.

- PID and ARSMC were used for comparison with the proposed method. In the single-leg joint control experiment, ARSMC and SMRC + UDE could significantly improve the control accuracy, while ARSMC exhibited the highest control accuracy among the three.
- Considering the performance of the control system hardware and the multiple IMHDUs (a total of 36 valves) of the hydraulic system, although ARSMC demonstrated superior control behavior, the dilemma between control performance and efficiency made it difficult to apply to our HHR. Thus, the proposed SMRC + UDE may be more suitable for achieving joint position control with high accuracy for the HHR, using only the directional control valves with lower cost.
- In the walking experiments, compared with PID, the MAXE of the knee and hip joints using SMRC + UDE in a quadrangular gait could reach 1.10° and 1.79°, with error reductions of 62.1% and 64.2%, respectively. In a tripod gait, the MAXE could reach 1.10° and 1.79°, with error reductions of 62.1% and 64.2%, respectively. Based on the characteristics of the repetitive control, the proposed SMRC + UDE demonstrated an excellent control performance when tracking the periodic gait (tripod and quadrangular gait).

Currently, there are diverse unstructured terrains to which legged robots must adapt, which introduces new demands for gait planning and controller design. Thus, exploring the potential for the proposed controller deployment in such nonperiodic free gaits is challenging. Future work will also focus on dealing with the oscillation in the stance phase and improving the energy efficiency based on the TSS and IMHDU. Furthermore, research on active compliance control, such as impedance control and virtual model control, as well as on some energy consumption optimization strategies, will be conducted.

## Figures and Tables

**Table 1:** Valve configuration in different phases.

$\mathrm{Ground}\;\mathrm{Reaction}\;\mathrm{Force}\;G_{F}$	Phase	$\mathrm{Valve}\;V_{v1}$	$\mathrm{Valve}\;V_{v2}$	$\mathrm{Valve}\;V_{v3}$
$G_{F}\geq 100$	Stance phase	On	Off	On
$G_{F}<100$	Swing phase	Off	On	On

**Table 2:** The comparison of three control algorithms regarding control accuracy and CPU runtime in E2.

Control Algorithm	MAXE (°)		RMSE (°)		Runtime (ms)
	$\theta_{1}$	$\theta_{2}$	$\theta_{1}$	$\theta_{2}$	
PID	8.71	9.57	3.64	3.67	0.25
ARSMC	5.83	4.34	2.18	1.62	2.04
SMRC + UDE	6.02	4.78	2.27	1.78	0.90

## Data Availability

All the data presented in this study are available in the main text.

## Appendix A

**Lemma A1.** 
*Considering the system Equation (41) and the filter Equation (43), let an auxiliary variable* $\beta =\frac{\left(z_{2}-z_{2f}\right)}{T_{s}}-p_{1}u_{1f}-\zeta_{1}$ *be bounded and decrease to a small residual set exponentially for* $T_{s}>0$ *. Hence, the manifold* β=0 *is an invariant manifold for* $T_{s}\to 0$ *.*

**Proof of Lemma A1.** The derivative of β can be calculated as

(A1)
$$\begin{array}{cc} \dot{\beta } & =\frac{\left({\dot{z}}_{2}-{\dot{z}}_{2f}\right)}{T_{s}}-p_{1}{\dot{u}}_{1f}-{\dot{\zeta }}_{1}\\ & =\frac{\zeta_{1}+p_{1}(u_{1}-T_{s}{\dot{u}}_{1f})-{\dot{z}}_{2f}}{T_{s}}-{\dot{\zeta }}_{1}\\ & =\frac{\zeta_{1}+p_{1}u_{1f}-\frac{\left(z_{2}-z_{2f}\right)}{T_{s}}}{T_{s}}-{\dot{\zeta }}_{1}\\ & =-\frac{\beta }{T_{s}}-{\dot{\zeta }}_{1} \end{array}$$

A positive semi-definite Lyapunov function can be chosen as $V=\frac{1}{2}\beta^{2}$, and based on Young’s inequality, it can be derived that

(A2)
$$\dot{V}=\beta (-\frac{\beta }{T_{s}}-{\dot{\zeta }}_{1})\leq -\frac{\beta^{2}}{T_{s}}+\frac{1}{2}(\frac{\beta^{2}}{T_{s}}+T_{s}{{\dot{\zeta }}_{1}}^{2})\leq -\frac{V}{T_{s}}+\frac{T_{s}{ƛ}^{2}}{2}$$

Thus, we can verify that

(A3)
$$V\leq e^{-\frac{t}{k}}V(0)+\frac{k^{2}{ƛ}^{2}}{2}[1-e^{-\frac{t}{k}}]\leq e^{-\frac{t}{k}}V(0)+\frac{k^{2}{ƛ}^{2}}{2}$$

(A4)
$$\left|\beta (t)\right|=\sqrt{2V}\leq \sqrt{\beta^{2}(0)e^{-\frac{t}{k}}+k^{2}{ƛ}^{2}}$$

This implies that the exponential boundedness of β for $T_{s}>0$ and the manifold β=0 is an invariant manifold for $T_{s}\to 0$. □

## Appendix B

The main parameters of the controllers in the prototype experiments are shown in Table A1.

**Table A1:** Main parameters of the controllers in the prototype experiments.

Controller	Gain	Value		
		Root	Hip	Knee
PID	$k_{p}$	50	200	200
	$k_{i}$	0.1	0.1	0.1
	$k_{d}$	1	1	1
ARSMC	$k_{0}$	-	500	1000
	$k_{1}$	-	0.5	1
	$k_{2}$	-	0.0001	0.0001
	ρ	-	1	1
	ε	-	1000	1000
	Γ	-	diag(1,1,1,1,1,1)	diag(1,1,1,1,1,1)
SMRC + UDE	$k_{0}$	-	500	1000
	$k_{1}$	-	0.5	1
	$k_{2}$	-	0.0001	0.0001
	k	-	0.01	0.01
	ρ	-	1	1
	ε	-	1000	1000
	L	-	0.001	0.001
	Q(s)	-	0.95	0.95
